# Identification and Characterization of the HD-Zip Gene Family and Dimerization Analysis of HB7 and HB12 in *Brassica napus* L.

**DOI:** 10.3390/genes13112139

**Published:** 2022-11-17

**Authors:** Xiangyong Peng, Di Wu, Xin Zhang, Qingwei Liu, Qiuli Lu, Min Song

**Affiliations:** School of Life Science, Qufu Normal University, Qufu 273165, China

**Keywords:** HD-Zip genes, *Brassica napus*, phylogenetic analysis, synteny, BnHB7/HB12, homo- or hetro-dimerization

## Abstract

Homeodomain-leucine zipper (HD-Zip) genes encode plant-specific transcription factors, which play important roles in plant growth, development, and response to environmental stress. These genes have not been fully studied in allopolyploid *Brassica napus*, an important kind of oil crop. In this study, 165 HD-Zip genes were identified in *B. napus* and classified into four subfamilies. If proteins belong to the same subfamily, they exhibit similarities in gene structure, motifs, and domain distribution patterns. BnHD-Zip genes were unevenly distributed in the An and Cn subgenomes. Whole genome triplication (WGT) events may be major mechanisms accounting for this gene family expansion. Orthologous gene analysis showed that the process of this gene family expansion was accompanied by domain loss. We further found three genes homologous to HB7 and three genes homologous to HB12, all induced by PEG, ABA, and NaCl treatment. HB7 could not form homodimers but could form heterodimers with HB12 based on yeast two-hybrid assays. The results of this study provide valuable information for further exploration of the HD-Zip gene family in *B. napus*.

## 1. Introduction

Homeodomain-leucine zipper (HD-Zip) genes only exist in the plant kingdom and were first found in *Arabidopsis thaliana* [1]. HD-Zips, acting as transcription factors, mainly harbor a conserved homeodomain (HD) and an adjacent leucine zipper (LZ) motif [2]. The former is responsible for binding to DNA, and the latter mediates protein dimerization [3,4]. HD-Zip genes can be further divided into four subfamilies according to their overall structural characteristics [5]. The proteins in subfamily I are simple and only contain the HD and Zip domains. Compared to subfamily I, subfamily II proteins have a conserved CPSCE (Cys, Pro, Ser, Cys, and Glu) motif, which is adjacent to the LZ in the C-terminal direction [6]. The START and SAD domains are present in both subfamilies III and IV [7]. The former is short for steroidogenic acute regulatory protein-related lipid transfer domain and the latter for the START-associated domain. Subfamily III proteins have an additional MEKHLA domain at the C-terminal compared to subfamily IV. The name of this domain is the abbreviation of methionine–glutamic–lysine–histidine–leucine–alanine [5].

The functions of certain members of the HD-Zip family have been studied using mutants or transgenic plants in *A. thaliana*, rice, or other plants [8,9]. Using bioinformatics methods, the HD-Zip family has been explored in many species. Most of these studies are related to the stress response. In *Oryza sativa* [10], *Glycine max* [8], *Chrysanthemum morifolium* [11], *Manihot esculenta* [12], *Brassica rapa* [13], *Setaria italica* [8], *Triticum aestivum* [14], *Nicotiana tabacum* [15], *Jatropha curcas* [16], *Manihot esculenta* [17], *Eucalyptus grandis* [18], *Capsicum annuum* [8], *Dendrobium officinale* [19], *Medicago truncatula* [8], and *Zea mays* [20,21], some HD-Zip genes are regulated by one or multiple abiotic stresses, including drought, salt, osmotic, and temperature stress. There are some reports related to plant growth and development. In *Malus domestica*, the HD-Zip genes of subfamily III are enhanced during adventitious bud regeneration [8]. The expression of some HD-Zip genes change during fruit ripening in *Musa acuminata* [22] and stem and leaf–bud development in *Prunus mume* [23]. Meanwhile, some AtHD-Zip genes play roles during embryogenesis [9].

*Brassica napus* (rapeseed) is a considerable oil crop planted worldwide. *B. napus* is an allotetraploid of the *Brassica* genus, and *B. rapa* and *B. oleracea* are its diploid ancestors [24]. Researchers also regard it as a model plant when studying allopolyploids. Little is known about the HD-Zip gene family in *B. napus*. Using bioinformatics methods, we identified the HD-Zip genes of this species and focused on the phylogeny, structural characteristics, chromosomal localization, and synteny of the family in rapeseed. In addition, the expression patterns under abiotic stress of three genes homologous to HB7 and three genes homologous to HB12, as well as their dimerization, were examined. These results provide insights for a more comprehensive understanding of the characterization of the HD-Zip gene family in rapeseed.

## 2. Materials and Methods

### 2.1. Identification of HD-Zip Genes in B. napus

We used 48 HD-Zip protein sequences of *A. thaliana* to BLASTp (E-value < 1 × 10^−5^) *B. napus* protein sequences (versions: Brana_Dar_V5) downloaded from the BRAD database (http://brassicadb.org, accessed on 5 April 2020. Now the website address was moved to http://brassicadb.cn, accessed on 7 January 2022) [25]. Meanwhile, we searched the *B. napus* protein database using HMMER 3.0 with the default parameters employing PF00046 (HD domain) and PF02183 (HALZ domain) (https://pfam.xfam.org/, accessed on 24 April 2020). The domains were identified using CDD (http://www.ncbi.nlm.nih.gov/Structure/cdd/wrpsb.cgi/, accessed on 28 April 2020). Finally, all of the identified genes were renamed according to *Brassica* standard nomenclature [26]. In addition, we identified HD-Zips in eight varieties, including ZS11, Gangan, Zheyou7, Shengli, Tapidor, Quinta, Westar, and No2127, and protein sequences downloaded from the BnPIR database (http://cbi.hzau.edu.cn/cgi-bin/rape/, accessed on 15 October 2022). This gene family in *B. oleracea* (cv. Jinzaosheng) was also identified.

We used the ExPASy online tool to calculate each protein’s molecular weight (MW) and theoretical isoelectric point (pI), used Plant-mPLoc to predict the subcellular localization, and used the online PlantCARE tool to predict the cis-regulatory elements of the genes (1500 bp genomic sequences upstream before the initiation codon). The websites of the three tools are https://web.expasy.org/tools/ (accessed on 5 May 2020) http://www.csbio.sjtu.edu.cn/bioinf/plant-multi/ (accessed on 5 May 2020) and http://bioinformatics.psb.ugent.be/webtools/plantcare/html/ (accessed on 31 July 2020), respectively.

### 2.2. Phylogenetic Analysis

All HD-Zip protein sequences from *B. napus*, *B. rapa*, *B. oleracea*, and *A. thaliana* underwent multiple alignments using ClustalX1.8 [27]. An unrooted phylogenetic tree was constructed based on the neighbor-joining (NJ) method, with 1000 bootstrap replicates, using MEGA 7 software [28] and visualized using Evolview (https://www.evolgenius.info/evolview/, accessed on 9 April 2022) [29].

### 2.3. Gene Structure and Conserved Motif Analysis

The exon–intron structures of the BnHD-Zip genes were determined with TBtools [30] according to the GFF3 annotation information. The conserved motifs of the HD-Zip proteins were investigated with the MEME tool (http://meme-suite.org/, accessed on 22 August 2020). We downloaded the mast.xml result file and used the Redraw motif pattern tool of TBtools to display the distribution of the motifs.

### 2.4. Chromosomal Locations, Synteny, and Gene Replication Analysis

The chromosomal locations of the HD-Zip genes in three *Brassica* species were obtained according to the GFF3 annotation information and were displayed using Mapchart software [31]. MCscanX software was used to analyze the gene duplication events [32]. The synteny relationship of all HD-Zip genes from three *Brassica* species and *A. thaliana* was evaluated by searching “syntenic genes” in BRAD. The ratio of non-synonymous to synonymous substitutions (Ka/Ks) of orthologous pairs was calculated by TBtools [30].

### 2.5. Expression Analysis from RNA-Seq Data

The NCBI SRA dataset numbered SRP109808 was downloaded, which is the RNA-seq results of seven-day-old *B. napus* seedlings treated with 15% PEG-6000, 150 mM NaCl, and 30 μM ABA for 12 h [33]. We calculated the FPKM using Kallisto, and used TBtools to draw heatmaps of the expression levels of BnHD-Zip genes.

### 2.6. Plant Materials, Growth Conditions, and Treatments

Seeds of *B. napus* cv. ‘Zhongshuang 11’ were germinated on moist filter paper for 24 h at around 22 °C. Subsequently, they were transferred to a 1/2 Hoagland nutrient solution and grown at around 22 °C with a 16 h light/8 h dark cycle. After three weeks, the seedlings were treated with solutions containing PEG-6000, NaCl, and ABA, respectively. The concentrations were consistent with the description in Section 2.5. We collected leaves 0, 1, 3, 6, and 12 h after each treatment, froze them immediately with liquid nitrogen, and store them at −80 °C.

### 2.7. Quantitative Real-Time PCR

The kits used in this study were from Vazyme Biotech Co., Ltd. (Nanjing, China), unless otherwise specified. Total RNA was extracted using an EasyPure Plant RNA Kit (TransGen Biotech Co., Ltd., Beijing, China). The cDNA was synthesized using HiScript II Q RT SuperMix for qPCR with a gDNA wiper. Quantitative real-time PCR (qPCR) was carried out on a LightCycler 480 instrument (Roche LC 480, Roche, Basel, Switzerland), using a ChamQ SYBR Color qPCR Master Mix. All reactions were performed in triplicate. The reference gene (*BnActin*) was used as the internal control, based on the 2^−ΔΔCt^ method [34]. The primer sequences in this study are listed in Table S1. 

### 2.8. Subcellular Localization

The coding sequences of *BnaA.HB7*, *BnaC.HB7.a*, *BnaC.HB7.b*, *BnaA.HB12.a*, *BnaC.HB12.a*, and *BnaC.HB12.b* were cloned into the TA/Blunt-Zero cloning vector. The coding sequences of these genes were inserted sequentially into the *Xba* I and *BamH* I sites of the 35S_GFP_NOS_1300 vector with a ClonExpress II One Step Cloning Kit. The recombinant plasmid and the empty 35S_GFP_NOS_1300 were introduced into tobacco cells [35]. GFP fluorescence was observed via confocal laser scanning microscopy (Zeiss, LSM880 NLO, Carl Zeiss AG, Oberkochen, Germany).

### 2.9. Yeast Two-Hybrid (Y2H) Assay

The coding sequences of *BnaA.HB7*, *BnaC.HB7.a*, *BnaC.HB7.b*, *BnaA.HB12.a*, *BnaC.HB12.a*, and *BnaC.HB12.b* were inserted sequentially into the *EcoR* I and *Pst* I sites of pGBKT7 as prey and the *EcoR* I and *BamH* I sites of the pGADT7 vector as bait. Y2H assays were performed as previously described [36].

## 3. Results

### 3.1. Identification and Phylogenetic Analyses of HD-Zip Proteins

We used AtHD-Zip protein sequences to BLASTp protein sequences of *B. napus* and *B. oleracea*; meanwhile, an HMM-based search was performed with the HD and HALZ domains. As a result, 374 and 108 nonredundant putative protein sequences of *B. napus* and *B. oleracea* were initially detected, respectively. Then, those proteins without complete conserved characteristic domains were removed after searching in the SMART and CDD databases. Finally, 165 and 66 genes were identified as the HD-Zip genes in *B. napus* and *B. oleracea*, respectively. HD-Zips (88 genes) in *B. rapa* have previously been reported [13]. These identified HD-Zip genes in the three species were renamed (Table S2) [25]. Notably, no orthologs of *HDG6* or *HDG10* in *A. thaliana* were found in any of the three species.

**Figure 1:**
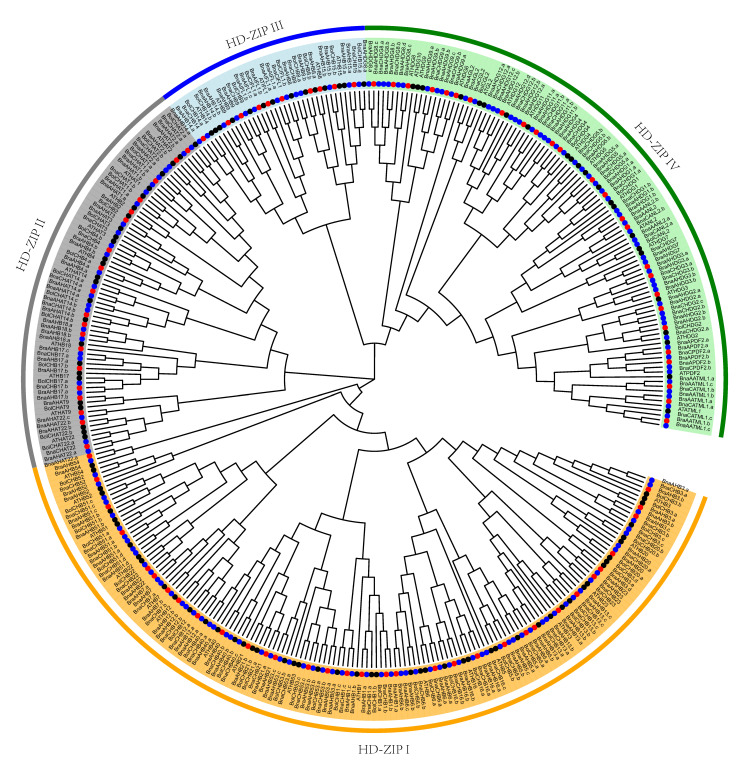
Phylogenetic analysis of HD-Zip proteins from *B. napus*, *A. thiliana*, *B. rapa*, and *B. oleracea*. This NJ phylogenetic unrooted tree was constructed using MEGA 7.0 and was divided into four subfamilies. Bootstrap replicates = 1000. The four species are represented with different circles in red (*B. napus*), blue (*B. rapa*), green (*B. oleracea*), and black (*A. thiliana*). There were 129 closely related sister pairs at the end of the branch of the phylogenetic tree, of which 63 were direct homologous pairs of the Ar–An genomes and 39 were Co–Cn pairs.

A total of 367 HD-Zip protein sequences from *A. thaliana* and the three *Brassica* species were used to construct an unrooted phylogenetic tree (Figure 1). Evidently, the HD-Zip proteins were classified into four subfamilies based on the phylogenetic tree. Subfamily I contained the most members (153 members), followed by subfamily IV (106), while subfamily III had only 37 members. Subfamilies I–IV contained 69, 26, 13, and 57 HD-Zip proteins of *B. napus*, respectively. In this study, three genes (BnaC03g55030D, BnaAnng30670D, and BnaC03g69040D) were not identified as HD-Zip III genes due to the lack of MEKHLA domain, although they were considered as HD-Zip III genes in previous studies [37]. They can be considered as HD-Zip III likely genes. The HD-Zips in *A. thaliana* and the orthologs in *Brassica* were clustered in the phylogenetic tree. This suggests that HD-Zip transcription factors are evolutionarily very closely related in these species. There were 129 closely related sister pairs at the end of the branch of the phylogenetic tree, of which 63 were direct homologous pairs of the Ar–An genomes and 39 were Co–Cn pairs. A single phylogenetic tree of each subgroup was constructed to show a more high-resolution evolutionary process (Figure S1). Amino acid alignments for four BnHD-Zip sub-families members are shown in Figure S2.

The length of protein sequences from subfamilies I, II, III, and IV in *B. napus* ranged from 156 to 311, 181 to 340, 738 to 850, and 393 to 830 amino acids, with molecular weights (MWs) varying between 17.91 and 35.94, 20.45 and 37.97, 81.35 and 93.05, and 44.42 and 92 KDa, as well as theoretical PI values varying between 4.54 and 9.21, 7.63 and 10.06, 5.62 and 6.15, and 5.24 and 8.63, respectively. Analysis of localization prediction ascertained that the HD-Zips were located in the nucleus. The MWs and theoretical PI values of the HD-Zips of the other two *Brassica* species were also analyzed and are provided in Table S1.

In addition, we identified HD-Zip genes in eight varieties, including ZS11, Gangan, Zheyou7, Shengli, Tapidor, Quinta, Westar, and No2127. The number of HD-Zip genes in each variety was different, and the variety ZS11 had the most genes, while Zheyou7 had the least numbers (Table 1). More information is shown in Table S3. In the study, the gene family analysis focused on the reference genome of Darmor.

### 3.2. Gene Structure and Conserved Motif Analysis

**Figure 2:**
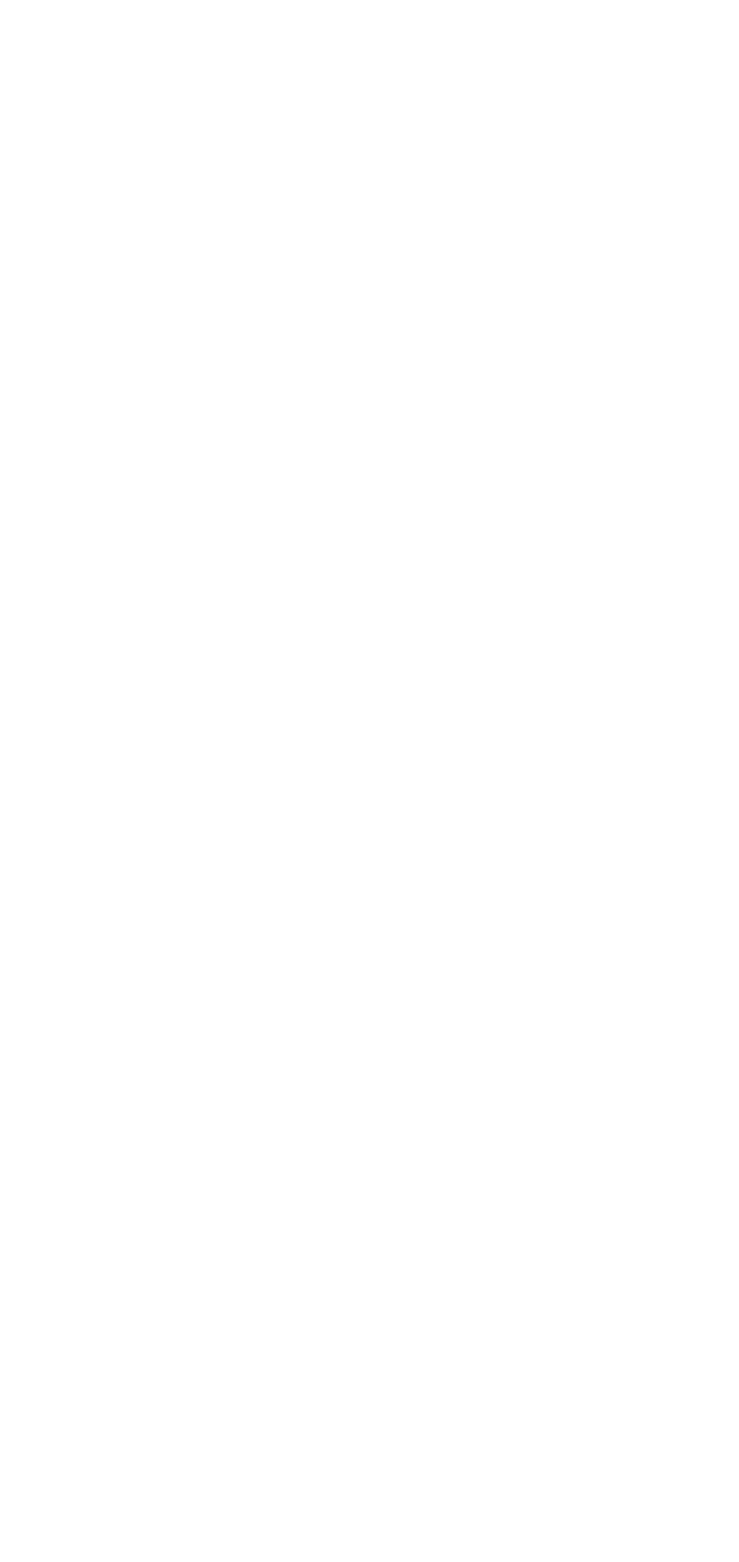
Phylogenetic tree (**Left**), motif distributions (**Medium**), and gene structures (**Right**) of the HD-Zip family in *B. napus.* The gene structures and motifs had a similar distribution in the same subfamily, and were more complex in subfamily III/IV than in subfamily I/II.

The number of introns and exon–intron arrangement showed obvious differences among the four HD-Zip subfamilies in *B. napus* (Figure 2). However, members of the same subfamily shared the same or similar exon–intron patterns. Genes of subfamilies I and II had zero to four introns. In subfamily I, approximately 91.3% of members had one to two introns. *BnaC.HB20.b* contained the most introns, while *BnaA.HB52* and *BnaC.HB52* lacked introns. Of the genes in subfamily II, 69.2% (18 genes) had three introns. The HD-Zip genes in subfamily III had 12–17 introns. Meanwhile, the HD-Zip genes in subfamily IV had 5–13 introns, while 64.9% of the genes had eight to nine introns. *BnaC.HDG2.b* had the lowest number of introns, while *BnaA.HDG2.b* and *BnaC.ATML1.c* had the most. In all, the gene structures were more complex in subfamily III/IV than in subfamily I/II.

In addition, the conserved motifs in the HD-Zips were searched using the online MEME tool. As Figure 2 shows, motifs 2-1-6 were found in most of the HD-Zip proteins, though some proteins lacked motif 2, such as BnaC.HB16.c and BnaA.HB18.b, or motif 6, such as BnaA.HDG8.b, BnaC.HDG8.a, and BnaA.HDG10.b. The HD domain spanned the sequence of motifs 2 and 1, and the LZ domain covered motif 6. The HD-Zip proteins of subfamily III added motifs 3 and 4, corresponding to the START domain, while the HD-Zip proteins of subfamily IV added motifs 8, 10, 5, 9, and 7 compared to the HD-Zip proteins in subfamily III. Motif 6 appeared twice in series in 64.9% of the HD-Zip proteins in subfamily IV. In general, the motifs had a similar distribution in the same subfamily.

### 3.3. Chromosomal Location and Gene Duplication Analysis

**Figure 3:**
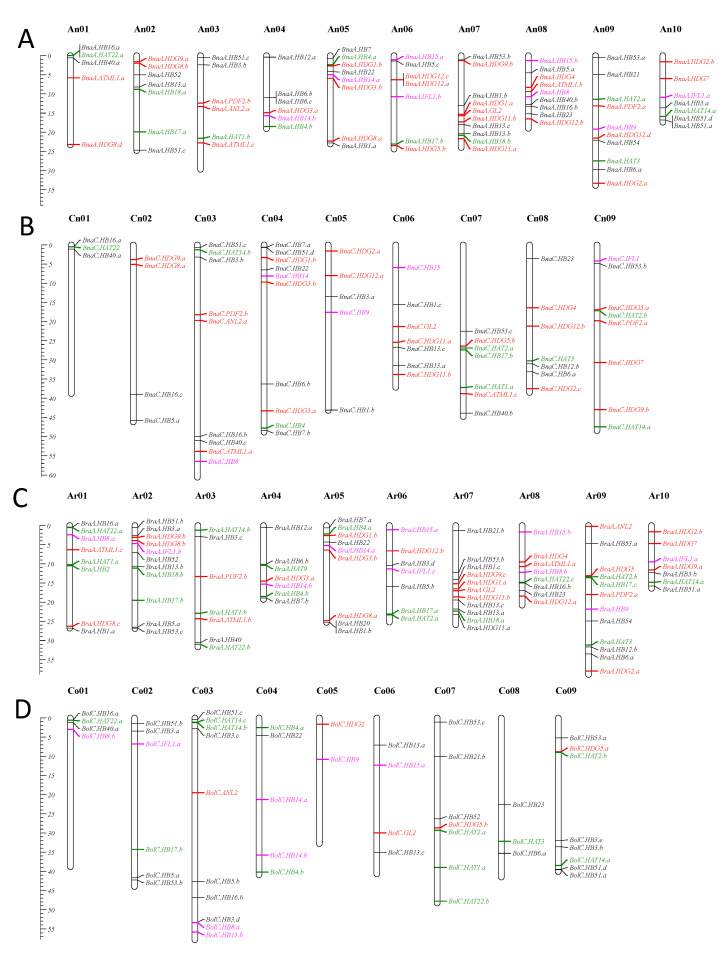
Chromosomal location of HD-Zip genes in *B. napus* (**A**,**B**), *B. rapa* (**C**), and *B. oleracea* (**D**). Partial HD-Zip genes are not shown in this figure due to being located in unassembled scaffolds. The scale on the left is in megabases (Mb). Genes in black, green, pink, and red represent genes belonging to subfamily I, II, III, and IV, respectively. These genes in the Ar and Co genomes were similar to those in the An and Cn subgenomes.

We acquired the position information of the BnHD-Zip genes based on the genome annotation information. It was found that 135 BnHD-Zip genes had clear chromosomal location information and were unevenly distributed on the chromosomes in *B. napus* (Figure 3A,B). There were 86 and 79 HD-Zip genes in the An and Cn subgenomes, respectively. Among the 30 genes not shown on the map, 25 genes were located in unanchored random regions and four genes in An03_random, An06_random, An09_random, and Cn02_random, respectively. Chromosomes An07 and An09 contained the most HD-Zip genes (10 genes), while Co01 contained the least (three genes).

In *B. rapa*, 87 BrHD-Zip genes were unevenly located on 10 chromosomes, except *BraA.HB21.a*, which was localized on the scaffold (Figure 3C). Chromosome Ar09 carried the most HD-Zip genes (12 genes), followed by 11 genes on Ar02 and Ar07. Chromosomes Ar03, Ar04, Ar06, and Ar10 each carried 7 genes.

In *B. oleracea*, 49 BoHD-Zip genes were distributed among nine chromosomes. The remaining 17 genes were located on scaffolds due to incomplete genome assembly (Figure 3D). Chromosome Co03 carried the most HD-Zip genes (12), while Co05 only contained two genes. After comparative analysis, we found that these genes in the Ar and Co genomes were similar to those in the An and Cn subgenomes, which may be attributed to *B. napus* being derived from hybridization between *B. rapa* and *B. oleracea*.

Among all of the 165 BnHD-Zips, 131 were derived from segmental duplication/WGD and two clusters of tandem repeat genes (*BnaA.HDG12.a/12.c* and *BnaA.HB51.a*/*51.d*) (Table S4). In *B. oleracea* and *B. rapa*, most of the BoHD-Zips (79.6%; 39/49) and BrHD-Zips (88.5%; 77/87) were also derived from segmental duplication/WGD (Table S4). Thus, segmental duplication/WGD appears to play an important role in the expansion of this family in the three *Brassica* species. Duplication types of genes located on unassembled scaffolds were not detected. It is worth mentioning that one cluster of HD-Zip tandem repeat genes was found in *B. oleracea*, but not in *B. napus*.

### 3.4. Synteny Gene Analysis of HD-Zip Genes

Using the “syntenic gene” tool in BRAD, we found that 44 of the 48 *A. thaliana* HD-Zip genes had corresponding syntenic genes in the three *Brassica* species (Table S5). There were 35, 24, and 19 BrHD-Zip genes, 21, 13, and 14 BolHD-Zip genes, and 49, 32, and 26 BnHD-Zip genes located in the LF, MF1, and MF2 subgenomes, respectively [38]. Most of the retained HD-Zip genes in the three species are located in the LF subgenome, with the least in the MF2 subgenome, reflecting the severe loss of orthologous genes. In addition, some syntenic genes of the AtHD-Zip genes were similar to those of the HD-Zip genes, but were not identified as HD-Zip genes because they lack complete characteristic domains, indicating that they might have lost certain domains during evolution.

The copy numbers of syntenic genes in each genome differed. There were 18 conserved genes retained from the Ar to An subgenomes, and 10 conserved genes retained from the Co to Cn subgenomes (Table S5). Meanwhile, the number of other genes retained in the An or Cn genome did not exceed the number of genes in the corresponding Ar or Co genome. For example, three *A. thaliana* HD-Zip genes (*ATHB20*, *ATHAT9*, and *ATHB2*) lacked syntenic genes in *B. napus*, but these genes were retained in the other two species, indicating that gene loss events happened during the evolution of *B. napus*.

To comprehend whether natural selection acted on the evolution of the HD-Zip gene family in *B. napus*, selection pressure analysis was performed on the HD-Zip orthologous pairs. The Ka/Ks ratios of the orthologous gene pairs were shown in Table S6. The Ka/Ks ratios of *BnaA.HB13.c*-*BnaC.HB13.c*, *BnaC.HB5.a*-*BolC.HB5.a*, and *BnaC.HAT22*-*BolC.HAT22.a*, were larger than 1, which indicated that these two genes were subject to positive selection pressure. The rest genes had Ka/Ks ratios that <1, which indicated that they underwent purifying selection during the evolution process and may preferentially conserve function, with mutations being disadvantageous.

### 3.5. The Promoter of BnHD-Zip Genes

**Figure 4:**
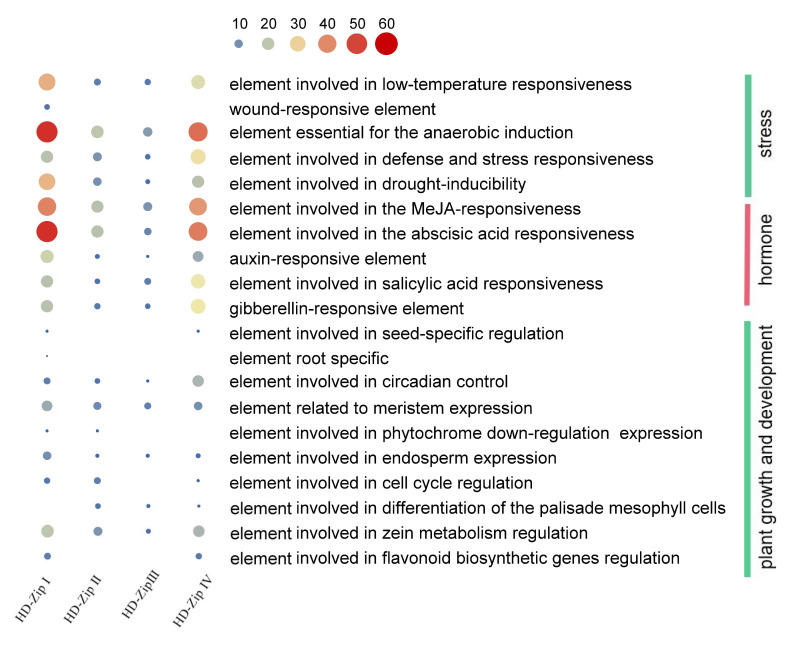
Overview of the number of BnHD-Zip genes with the main cis-acting elements on promoters in four subfamilies.

A total of 3705 diverse elements were found upstream of the BnHD-Zips. The names and functions of each element of certain genes are shown in Table S7. The HD-Zip promoter contained a large number of light response elements, plant growth- and development-related elements, hormone response elements, and stress response elements, in addition to TATA-box, CAAT-box, and unknown function elements (Figure 4). The elements were numbered from 1 (*BnaA.HB20*) to 43 (*BnaC.HB7.b*) and the type number from 1 (*BnaA.HB20*) to 20 (*BnaC.HB7.b* and *BnaC.HB1.a*). More than 50% of the genes in each subfamily had light-responsive elements, including G-box, Box 4, GT1-motif and TCT-motif, anaerobic induction elements, abscisic acid-responsive elements, and MeJA responsive elements. In addition, more than half of the subfamily I genes contained low-temperature responsive elements and more than half of the subfamily III genes contained SA responsive elements.

### 3.6. Abiotic Stress Expression Analysis

We analyzed the expression pattern of BnHD-Zip genes after treatment with ABA, PEG, and NaCl based on RNA-seq data (GEO:SRP109808) [33]. The FPKM values of 43 HD-Zip genes were not lower than 2 in three repeats of at least one treatment or control (Figure S3). A value of log_2_ FC ≥ 1 means that the gene expression increased; FC means the ratio of the value of any repeats in the treatment group to the value of any repeats in the control group. The results showed that the expression level of nine, three, and one subfamily I genes was upregulated after treatment with ABA, NaCl, and PEG, respectively (Figure S3).

**Figure 5:**
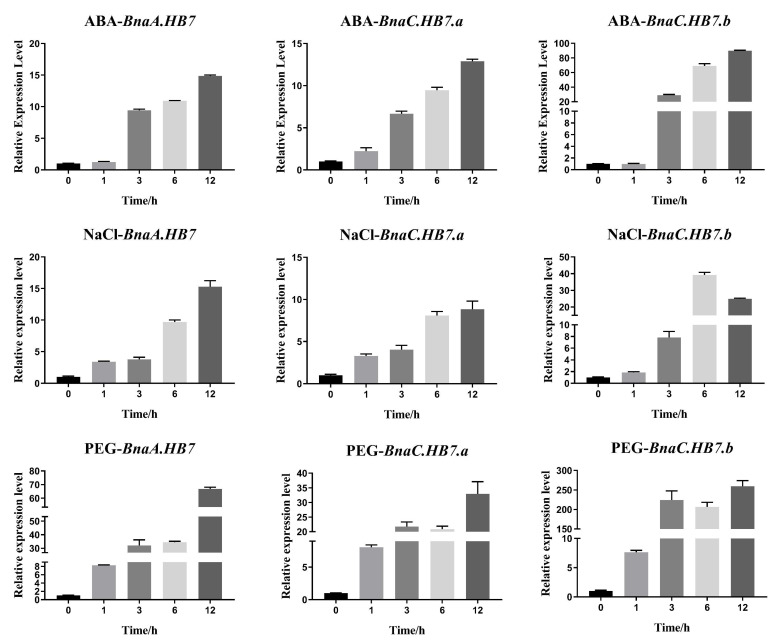
The expression level of *BnaA.HB7*, *BnaC.HB7.a*, and *BnaC.HB7* after treatment with ABA, NaCl, and PEG. *BnActin* was used as the internal reference. The genes were all upregulated after these treatments.

Furthermore, we selected three homologs of HB7 and HB12 to investigate their responses to the three stresses using qRT-PCR. As a homologous group, *BnaA.HB7*, *BnaC.HB7.a*, and *BnaC.HB7.b* showed similar stress responses, being increased by the ABA, NaCl, and PEG treatments (Figure 5). They reached peak expression levels at 12 h, except for *BnaC.HB7.b*, which reached a peak at 6 h under NaCl treatment. *BnaC.HB7.b* increased nearly 100-fold compared to the control.

**Figure 6:**
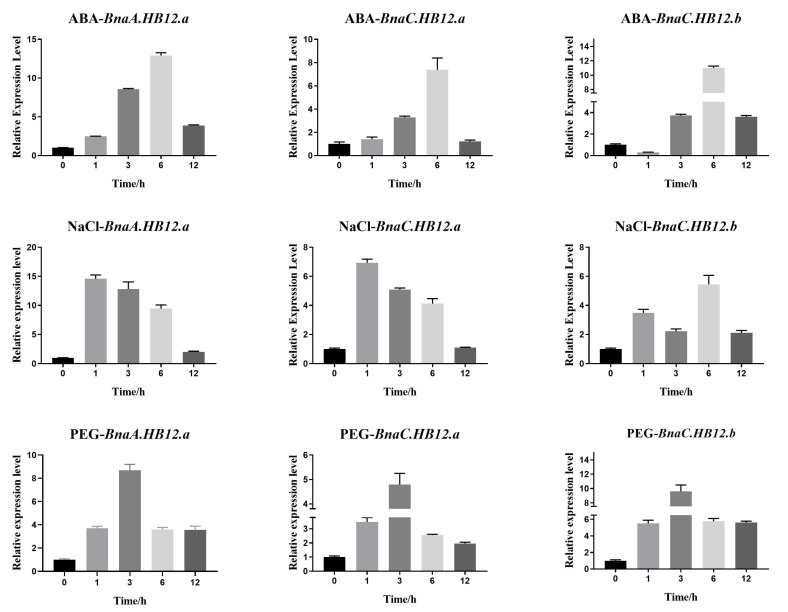
The expression level of *BnaA.HB12.a*, *BnaC.HB12.a*, and *BnaC.HB12.b* after treatment with ABA, NaCl, and PEG. *BnActin* was used as the internal reference. The genes were all upregulated after these treatments.

Members of the HB12 homologous group—*BnaA.HB12.a*, *BnaC.HB12.a*, and *BnaC.HB12.b*—also showed similar stress responses, being upregulated by these treatments (Figure 6). Unlike HB7 genes, *BnaA.HB12.a*, *BnaC.HB12.a*, and *BnaC.HB12.b* reached a peak after 6 h ABA treatment and 3 h PEG treatment, respectively. They reached peak expression levels at 1 h, except for *BnaC.HB12.b*, which reached a peak at 6 h after NaCl treatment. Overall, *BnaA.HB7*, *BnaC.HB7.a*, *BnaC.HB7.b*, *BnaA.HB12.a*, *BnaC.HB12.a*, and *BnaC.HB12.b* were all increased upon exposure to the three stresses.

### 3.7. Subcellular Localization Analysis

**Figure 7:**
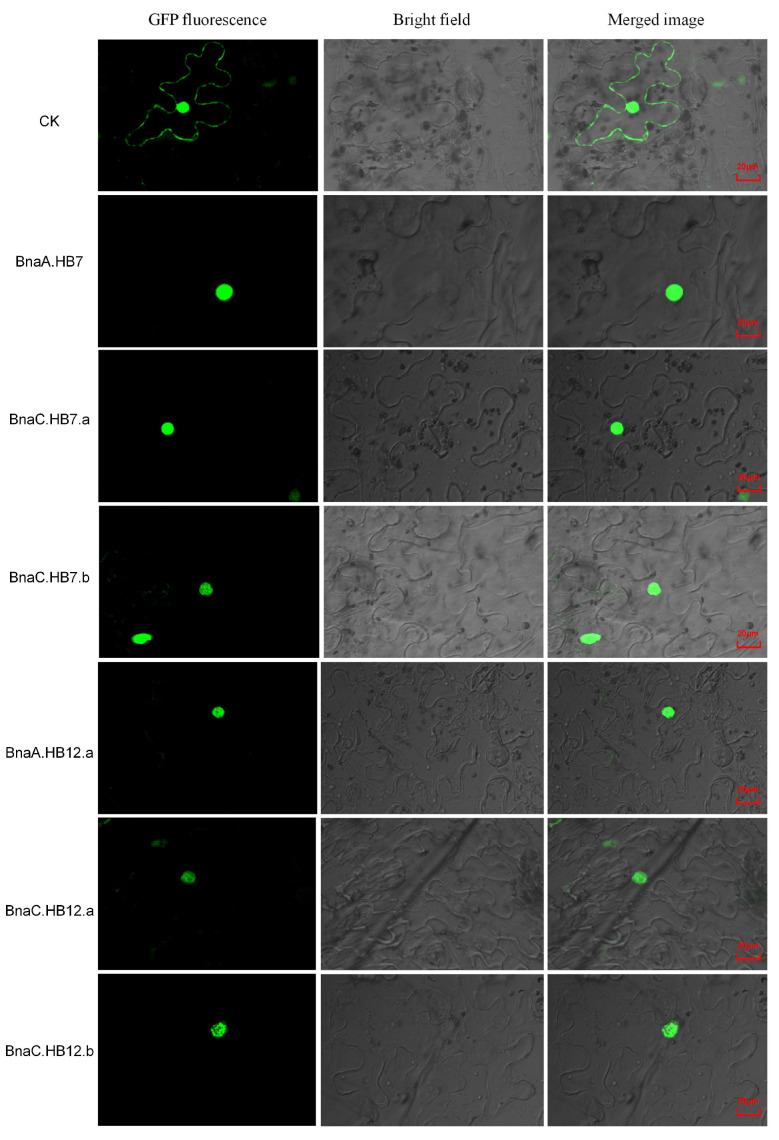
Subcellular localization of six BnHD-Zip proteins. Green fluorescence images were taken in a dark field, while the outline of the cell was taken in a bright field. CK: 35S: GFP represents the control.

To identify the subcellular localization of BnaA.HB7, BnaC.HB7.a, BnaC.HB7.b, BnaA.HB12.a, BnaC.HB12.a, and BnaC.HB12.b, we constructed their transient expression vectors fused with GFP. As shown in Figure 7, the fluorescent signal of these proteins was detected specifically in the nucleus, indicating nuclear localization of the six proteins, consistent with bioinformatics predictions.

### 3.8. BnHB7 and BnHB12 Might Act in Homo- or Heterodimers

**Figure 8:**
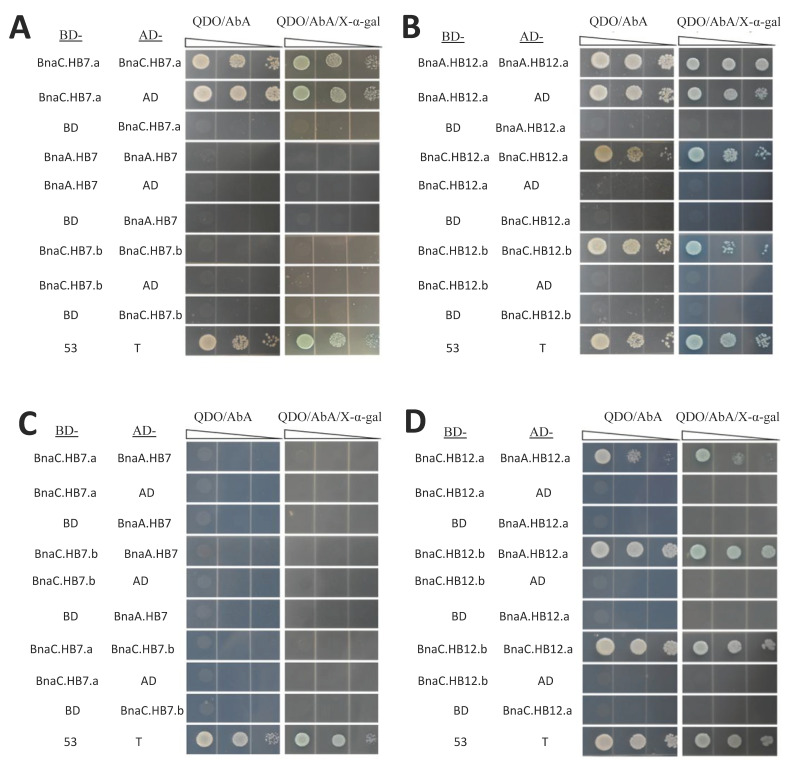
Results of the protein–protein interaction assay among the homologs of BnHB7 (**A**,**C**) or BnHB12 (**B**,**D**) by a yeast two-hybrid assay. QDO/AbA: Synthetic medium lacking Leu, Trp, His, and Ade with AbA. QDO/AbA/X-α-gal: Synthetic medium lacking Leu, Trp, His, and Ade with AbA and X-α-gal. Any two of the three homologs of HB12 (BnaA.HB12.a, BnaC.HB12.a, and BnaC.HB12.b) could form homodimers in vitro, whereas HB7 showed the opposite.

**Figure 9:**
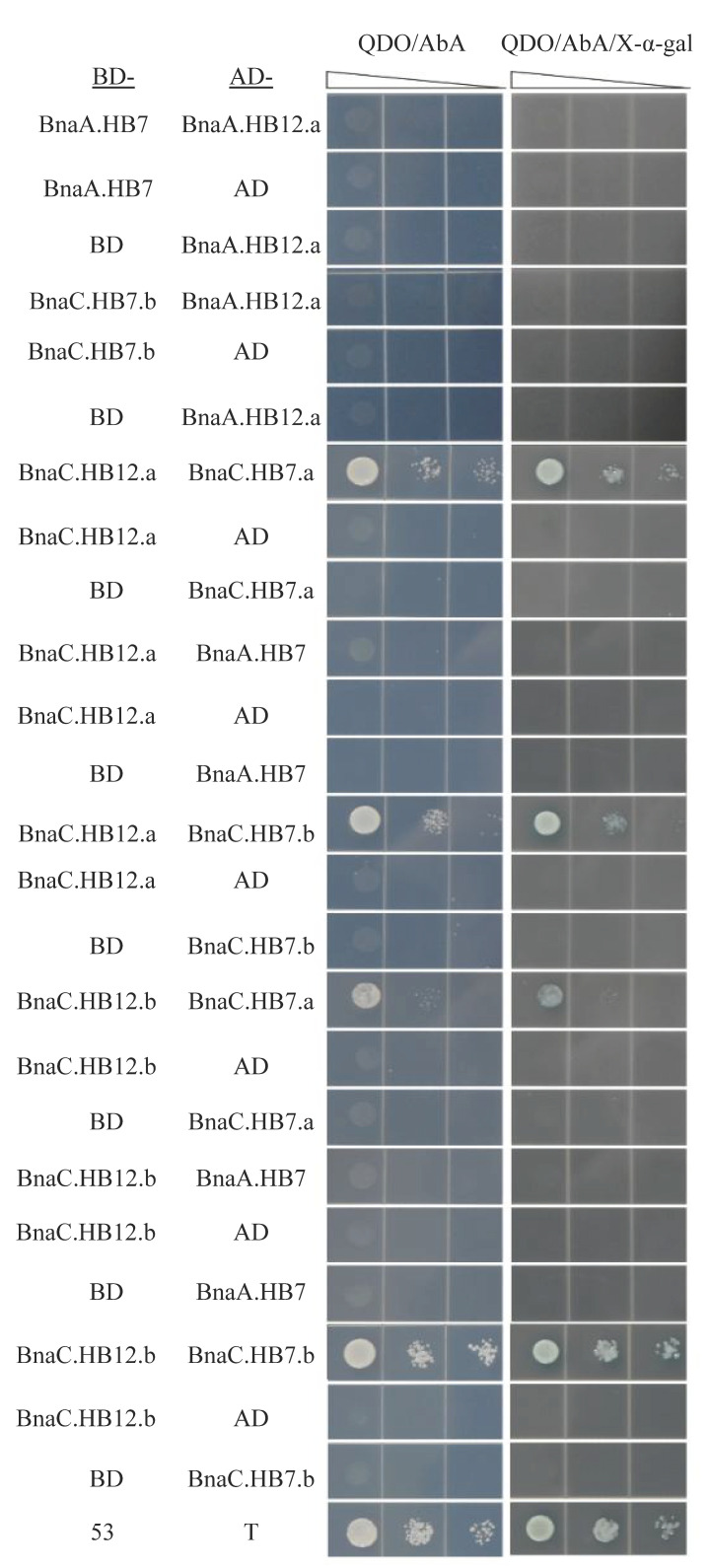
Results of the protein–protein interaction assay between BnHB7 and BnHB12 homologs by a yeast two-hybrid assay. Positive control: pGBKT7-53 as prey and pGADT7-T as bait. BnaC.HB12.a, similarly to BnaC.HB12.b, could form heterodimers with any BnaC.HB7.a and BnaC.HB7.b in vitro.

The leucine zipper domains in HD-Zip proteins could mediate the formation of protein dimers [8]. We detected interactions of the six proteins (BnaA.HB7, BnaC.HB7.a, BnaC.HB7.b, BnaA.HB12.a, BnaC.HB12.a, and BnaC.HB12.b) using a Y2H assay (Figure 8, Figure 9 and Figure S4). The results showed that some proteins could form homodimers with themselves, such as BnaC.HB12.a and BnaC.HB12.b. In contrast, BnaA.HB7 and BnaC.HB7.b could not form dimers with themselves. It is unclear whether BnaC.HB7.a and BnaA.HB12. can form a dimer with themselves, because the experimental results were the same as the control (Figure 8). Any two of the three homologs of HB12 (BnaA.HB12.a, BnaC.HB12.a, and BnaC.HB12.b) could form homodimers in vitro, whereas HB7 showed the opposite (Figure 8). Interestingly, BnaC.HB12.a, similarly to BnaC.HB12.b, could form heterodimers with any BnaC.HB7.a and BnaC.HB7.b in vitro (Figure 9). This suggests that there may be a cooperative relationship between them.

## 4. Discussion

The HD-Zip transcription factor has been identified in many plants. However, there are few reports on the family in rapeseed. *B. napus* formed approximately 7500 years ago through allopolyploidy, a process in which the ancestors *B. rapa* and *B. oleracea* crossed and then doubled their chromosomes [24]. *B. napus* has not only experienced a whole genome triploidization event (WGT) unique to *Brassica*, but also allopolyploidy, resulting in a huge genome of this species and driving gene family expansion. In this paper, 165 HD-Zip genes were identified in the *B. napus* genome, which is the highest number reported in plants so far, followed by soybean (101) [8]. Compared to its diploid progenitors, more putative HD-Zips in *B. napus* were identified, indicating that polyploidy has increased the number of HD-Zips in polyploid crops.

*Brassica* and *A. thaliana* differentiated from a common ancestor, and then the ancestor of *Brassica* experienced a WGT event. Therefore, in theory, the genes in *A. thaliana* should have three homologous genes in diploid *Brassica* and six homologous genes in allotetraploid *B. napus*. In fact, WGD led to the expansion of gene families and also affected the genome structure of *Brassica* [39]. After the separation of *Brassica* and *Arabidopsis* lineages, the doubling process led to the loss of *B. rapa* genome length. It was speculated that 35% of the existing genes were lost when the WGT event occurred in *Brassica* lineages [40]. In the present study, the number of HD-Zip genes obtained from two diploid *Brassica* was much less than three times that of *A. thaliana*, while the number of BnHD-Zip genes was much less than six times that of *A. thaliana.* We found that only 7 and 8 of 48 AtHD-Zip genes had three orthologous genes in the *B. rapa* and *B. oleracea* genomes, respectively. All of the AtHD-Zip genes had zero to two orthologs in the An or Cn subgenomes, apart from *AtHB13* and *AtATML1*, which had three homologs in the An subgenome. In addition, the syntenic genes of three AtHD-Zip genes had been deleted in *B. napus*, but they were retained in its two progenitors. These results indicate that the loss of HD-Zip genes occurred after the *Brassica* WGT event. It is noteworthy that 37 syntenic genes of AtHD-Zip in *B. oleracea* and six syntenic genes of AtHD-Zip in *B. rapa* were not identified as HD-Zip genes due to the lack of complete characteristic domains, indicating that domain loss events happened during the HD-Zip gene family expansion in the two species; in particular, the loss was more serious in *B. oleracea*. Synteny analysis revealed that most of the HD-Zip genes in the four species were located in conserved chromosomal blocks. Compared to *B. oleracea*, *B. rapa* lost more HD-Zip genes, which is consistent with the asymmetry of the gene loss between the two genomes.

The chromosomal distribution of the HD-Zip genes in the three *Brassica* species showed that HD-Zip orthologous genes are more similar in the *B. rapa* AA genome to the *B. napus* AA genome than in the *B. oleracea* CC genome to the *B. napus* CC genome, which may also be associated with more gene domain loss events during the expansion of the HD-Zip gene family in *B. oleracea* or the location of some genes are unclear. This is consistent with the findings for other gene families in the three species, such as GST [41], UGTs [42], and SUV [43]. A draft genome sequence of *B. oleracea*, comparing it with that of *B. rapa* reveals numerous chromosome rearrangements [38]. Chromosome rearrangement may be the main reason for the asymmetric distribution HD-Zip genes between the A and C sub-genome.

The phylogenetic tree showed that the HD-Zip genes of *B. napus* split into four subfamilies, and the domain characteristics of each subfamily protein showed a high degree of consistency in the same type of *A. thaliana*. Subfamily I has the most genes, while subfamily III has the least genes, which is consistent with the number of characteristics of the HD-Zip subfamilies of other plants. Among the 129 sister gene pairs, 102 were orthologous gene pairs formed by the subgenome of *B. napus* and the genome of their respective ancestral species, which indicates that the HD-Zip gene of *B. napus* is more closely related to the genes of its corresponding ancestral species than the homologous gene pairs.

HD-Zips are part of complex networks that integrate external signals by regulation of the hormone pathways involved in controlling basic developmental processes [44]. We found that many hormone response elements exist upstream of BnHD-Zip genes, including MeJA, ABA, GA, auxin, and SA—especially the ABA and MeJA hormone response elements (Table S4). Moreover, the patterns of elements showed no obvious differences between the four subfamilies. It is speculated that the HD-Zip family of *B. napus* may participate in these hormone-mediated plant signal regulatory networks and may play a further role.

Vast experimental evidence indicates that HD-Zip I subfamily proteins play an important role in plants’ response to stress [45]. *Arabidopsis AtHB7* and *AtHB12* genes are induced by ABA, drought, and salt stress [46]. The rice *OsHOX22*, homologous to *AtHB12*, protects against the adverse effects of long-term NaCl stress on plant survival [47]. We found that the homologous genes of *AtHB7* and *AtHB12* in *B. napus* were all upregulated by ABA, PEG, and NaCl treatment according to a qRT-PCR assay. However, the expression patterns differed between *BnHB7* and *BnHB12*. The abundance in the expression of the three homologs of *BnHB7* in the stress response was quite different, indicating that there may be dominant copies in executive function, and further genetic transformation experiments are needed.

**Figure 10:**
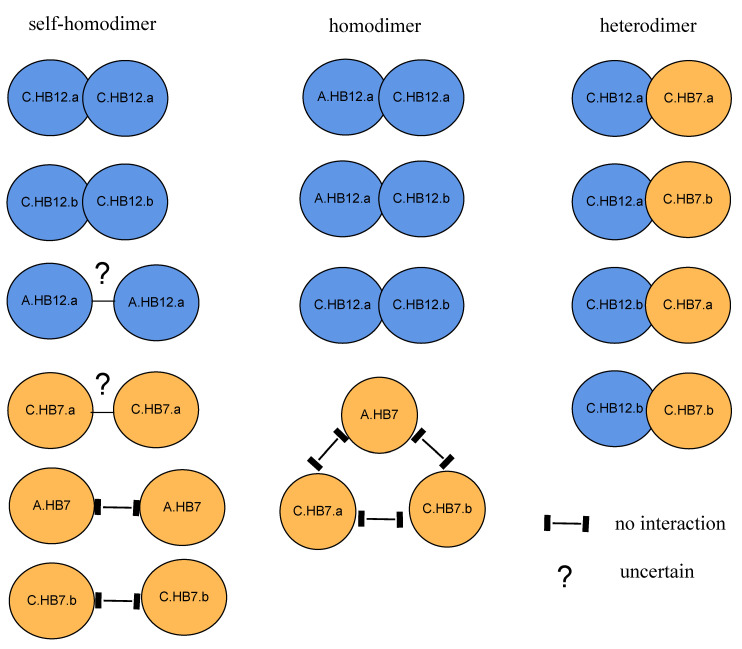
A summary of dimerization of BnaA.HB7, BnaC.HB7.a, BnaC.HB7.b, BnaA.HB12.a, BnaC.HB12.a, and BnaC.HB12.b in *B.napus.* “Bna” in the gene symbol was deleted for simplicity. Blue circles represent BnHB12 homologs and orange circles represent BnHB7 homologs. Positive control: pGBKT7-53 as prey and pGADT7-T as bait. The complex interaction pattern of homologs in *B. napus* may be due to the higher number of homologs.

The leucine zipper of HD-Zip proteins can realize the dimerization of HD-Zip proteins, which is a prerequisite for DNA binding [8]. In *A. thaliana*, AtHB5 can form homo- and heterodimers with other HD-Zip I members [48]. AtHB7 can not only form homodimers with itself, but can also form heterodimers with AtHB12 [36]. OsHox1, a rice HD-Zip II protein, can interact with the HAT, which is an *A. thaliana* HD-Zip I member [49]. Harris et al. [50] reported that three *T. asetivum* HD-Zip I members (TaHDZipI-3, TaHDZipI-4, and TaHDZipI-5) display interactions between one another and suggested a role for hetero-dimerization as a regulatory mechanism of their trans-activation function. In this study, we speculated that BnHB7 (BnaC.HB7.a and BnaC.HB7.b) and BnHB12 (BnaC.HB12.a and BnaC.HB12.b) may interact to form a heterodimer, while BnaA.HB7 cannot form a dimer with itself or with five other proteins, including BnaC.HB7.a, BnaC.HB7.b, BnaA.HB12.a, BnaC.HB12.a, and BnaC.HB12.b. On the contrary, BnaC.HB12.a and BnaC.HB12.b can form dimers with themselves or other proteins, except for BnaC.HB7.b. Overall, the more complex interaction pattern of homologs in *B. napus* may be due to the higher number of homologs compared to *A. thaliana* (Figure 10). Different interaction forms may regulate different target genes.

## 5. Conclusions

Conclusively, we identified 165 HD-Zip genes in *B. napus*, by far the largest number of identified genes in this family of species. Phylogenetic analysis showed that the HD-Zip proteins from *B. napus*, *A. thaliana*, *B. rapa*, and *B. oleracea* were divided into four subfamilies. Members of the same subfamily often have similar gene structures, motif distributions, and domain patterns. Phylogenetic and collinear analysis indicated that allopolyploidy was the main reason for rapeseed HD-Zip expansion. Additionally, the expression profiles in response to abiotic stress of the six genes and their dimerization between one another were analyzed. Together, these results can increase our understanding of the evolution of the HD-Zip gene family and lay a foundation for further understanding the role of HD-Zips in regulating abiotic stress in rapeseed.

## Figures and Tables

**Table 1:** Count of BnHD-Zip genes per variety.

Group	Darmor-bzh	Quinta	Tapidor	Shengli	Zheyou7	ZS11	Gangan	No2127	Westar
I	69	60	72	64	66	70	75	72	68
II	26	38	37	34	37	38	38	33	37
III	13	20	18	19	19	19	20	17	20
IV	57	50	52	52	34	53	42	51	46
Total	165	168	179	169	156	180	175	173	171

## Data Availability

Not applicable.

## Supplementary Materials

The following supporting information can be downloaded at: https://www.mdpi.com/article/10.3390/genes13112139/s1, Figure S1. Phylogenetic analysis of each HD-Zip subfamily from *B. napus*, *A. thiliana*, *B. rapa*, and *B. oleracea*. The NJ phylogenetic unrooted trees were constructed using MEGA 7.0; Figure S2. Amino acid alignments for BnHD-Zip members of each subfamily; Figure S3. An expression heatmap of BnHD-Zips under different stress was plotted according to the log2(FPKM + 1). BnHD-Zips with no or weak expression (FPKM < 2) in all samples were removed and the remaining family members were clustered by clades; Figure S4. Growth of double carrier co-transferred yeast in DDO medium. DDO: dropout medium without Leu and Trp; Table S1. Primer sequences in this study; Table S2. Information on the HD-Zip genes identified in the three species of *Brassica*; Table S3. HD-Zip genes identified in the eight *B. napus* varieties. ZS11, Gangan (GG), Zheyou7 (ZY), Shengli (SL), Tapidor (TA), Quinta (QU), Westar (WT), and No2127 (NO); Table S4. The four duplicated types of HD-Zip genes in the three species of *Brassica*; Table S5. Syntenic gene of HD-Zip genes in *A. thaliana* and the three species of *Brassica*; Table S6. Ka/Ks ratios of HD-Zip orthologous pairs; Table S7. The Cis-acting elements in the promoter regions of BnHD-Zip genes.
