# Supplementary material for: Identification and Characterization of the HD-Zip Gene Family and Dimerization Analysis of HB7 and HB12 in Brassica napus L
Source: Genes (Basel). 2022 Nov 17;13(11):2139. doi: 10.3390/genes13112139 (PMC9690955; doi:10.3390/genes13112139)
Supplement: Supplementary file 1 [file genes-13-02139-s001.zip › Figure S3.pdf]

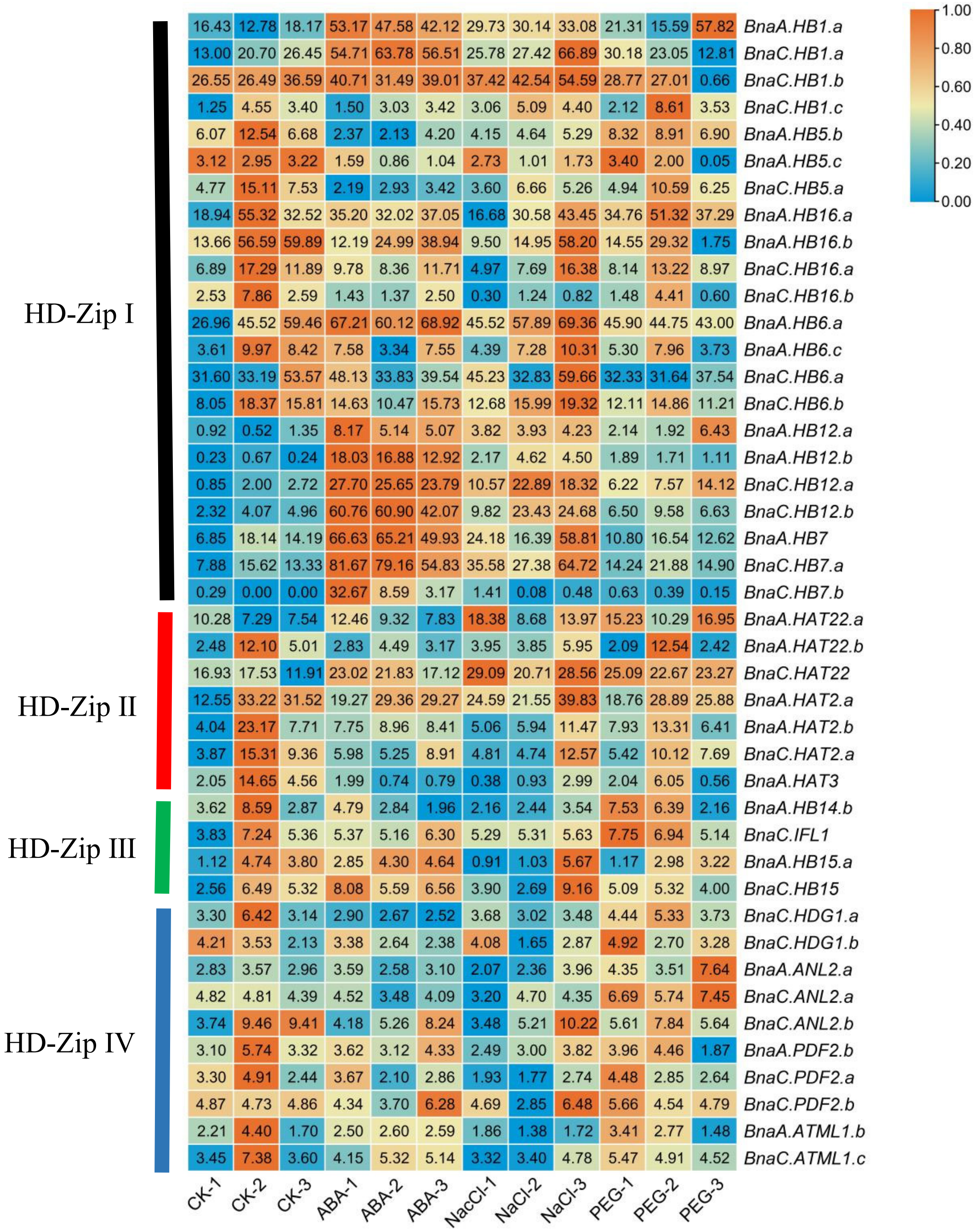

Figure S3. The expression heatmap of BnHD-Zips under different stress was plotted according to the log2 of (FPKM+1) in RNA-seq. BnHD-Zips with no or weak expression (FPKM < 2) in all samples were removed and the remaining family members were clustered by clades.
