# Supplementary figures and images for: Identification and Characterization of the HD-Zip Gene Family and Dimerization Analysis of HB7 and HB12 in Brassica napus L
Source: Genes (Basel). 2022 Nov 17;13(11):2139. doi: 10.3390/genes13112139 (PMC9690955; doi:10.3390/genes13112139)

# HD-Zip I

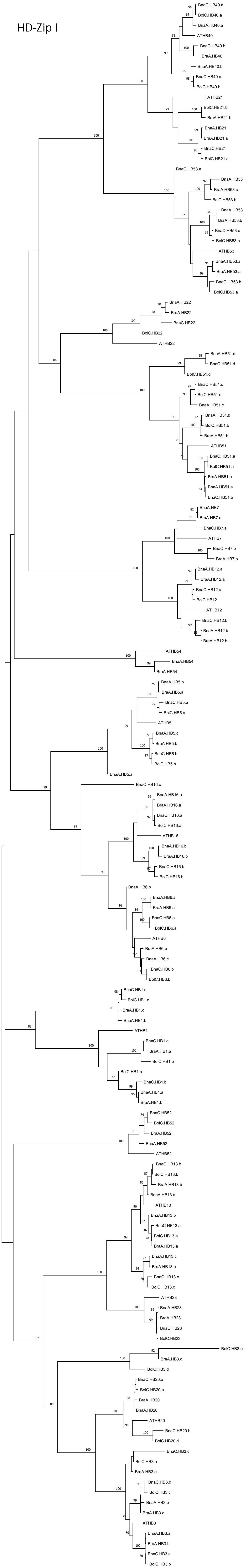

0.20

HD-Zip II

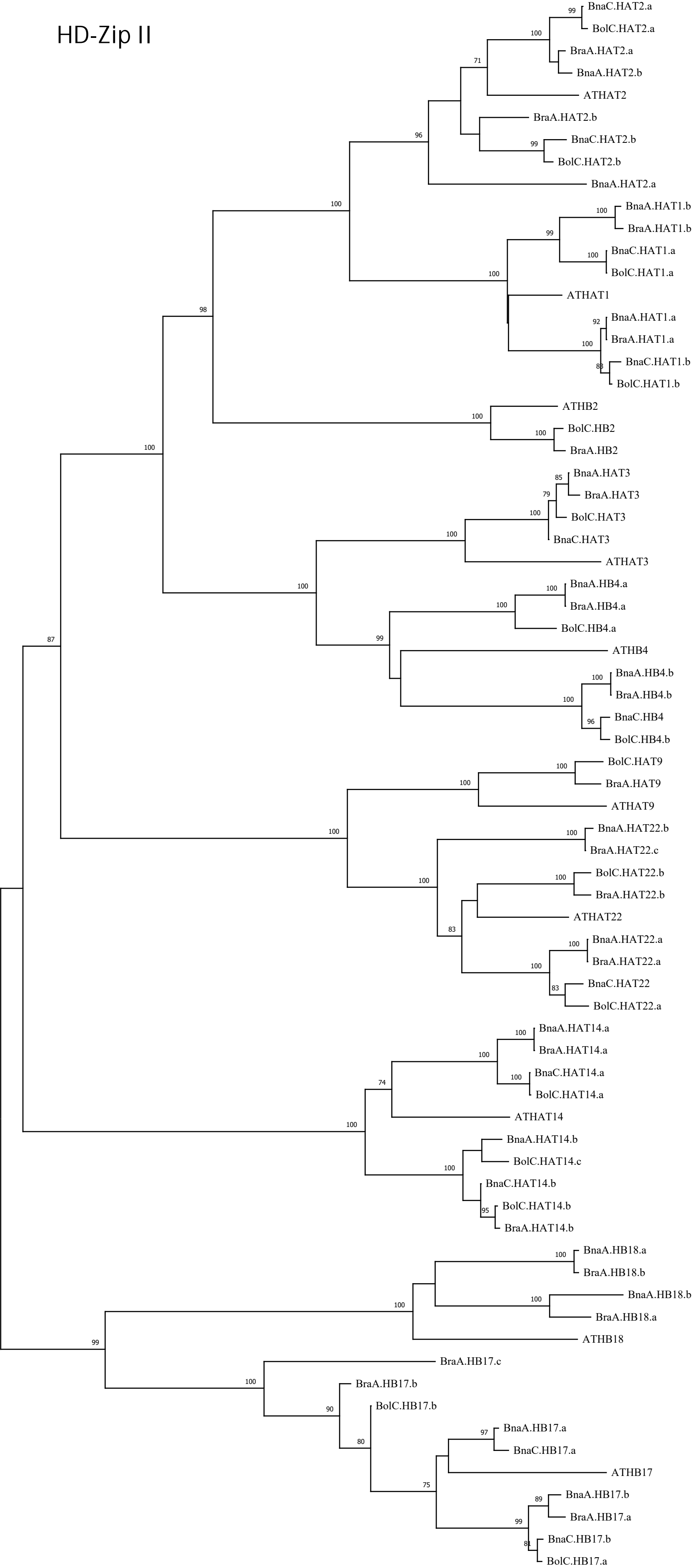

0.050

# HD-Zip III

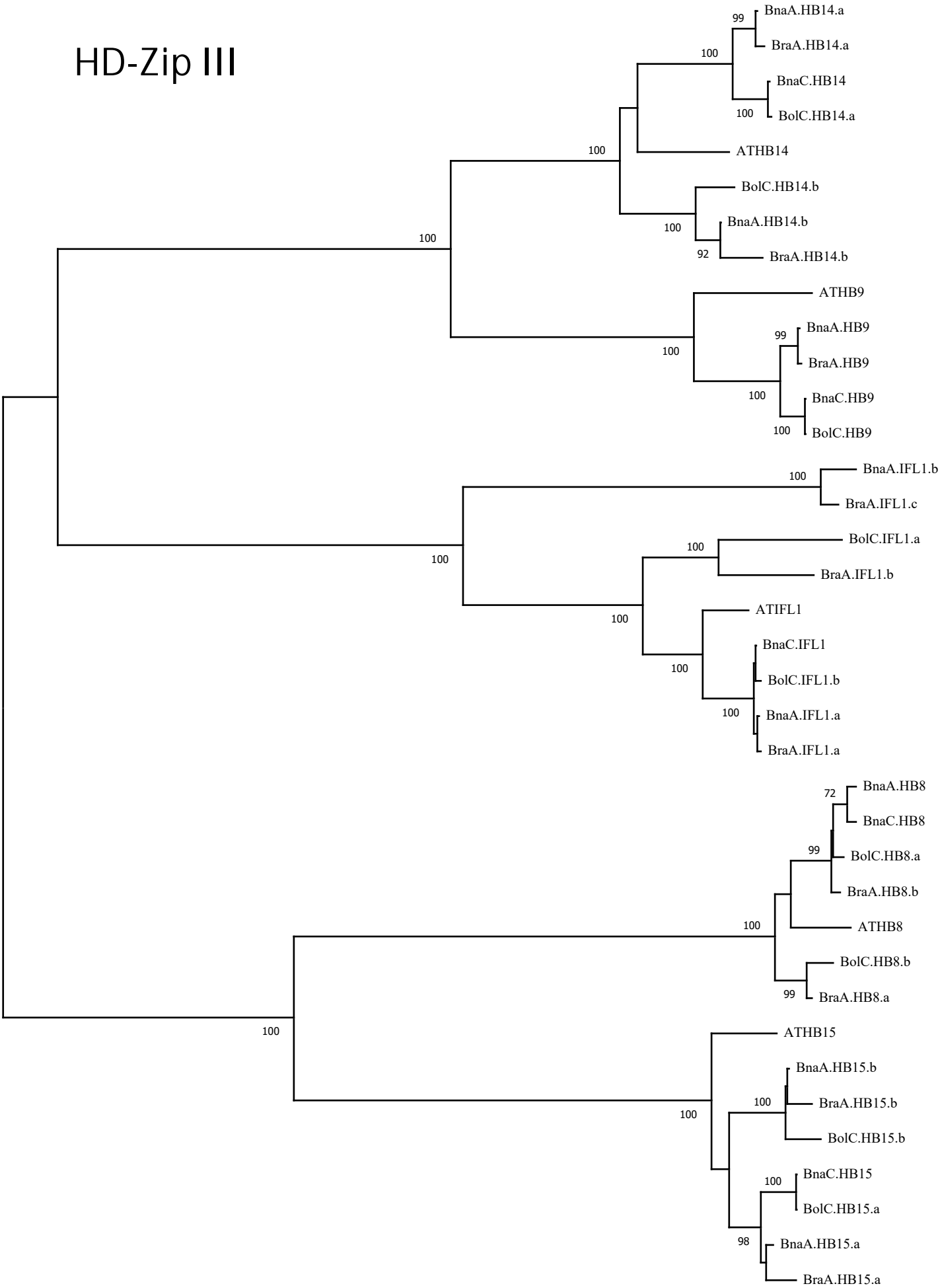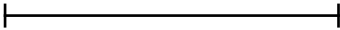

0.050

HD-Zip IV

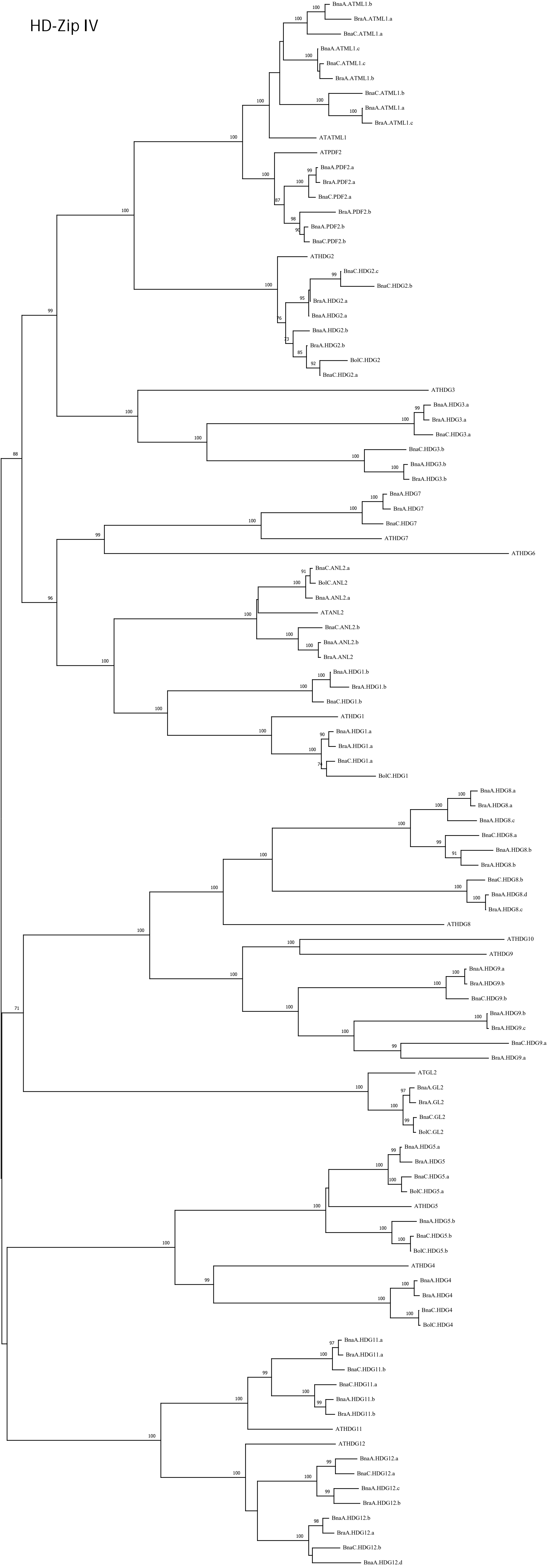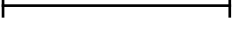

0.050

Supplement: Supplementary file 1 [file genes-13-02139-s001.zip › Figure S1.pdf]

## HD-Zip I

[illegible]

## HD-Zip II

[illegible]

## HD-Zip III

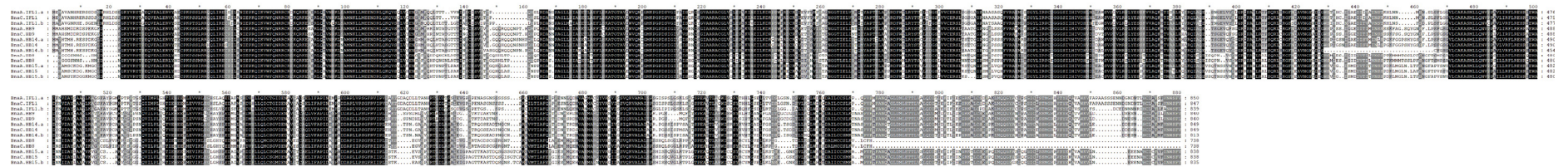

## HD-Zip IV

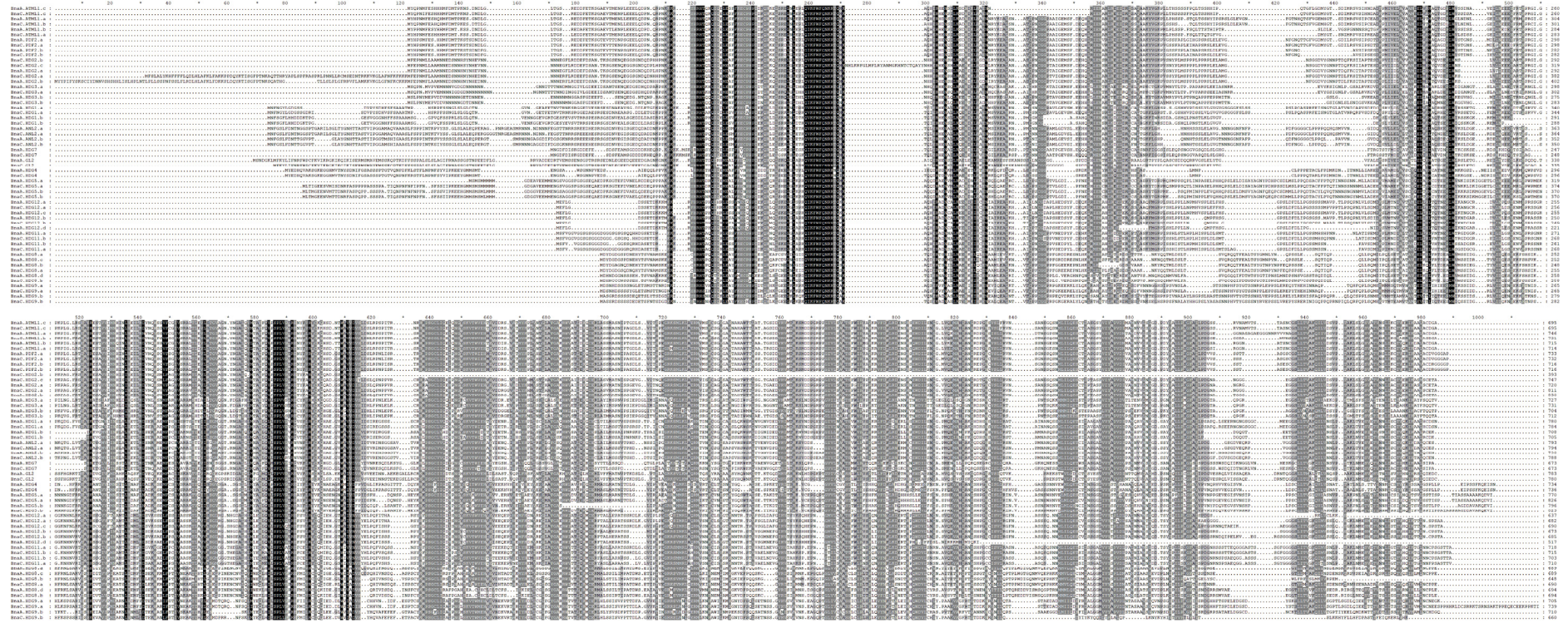

Supplement: Supplementary file 1 [file genes-13-02139-s001.zip › Figure S2.pdf]

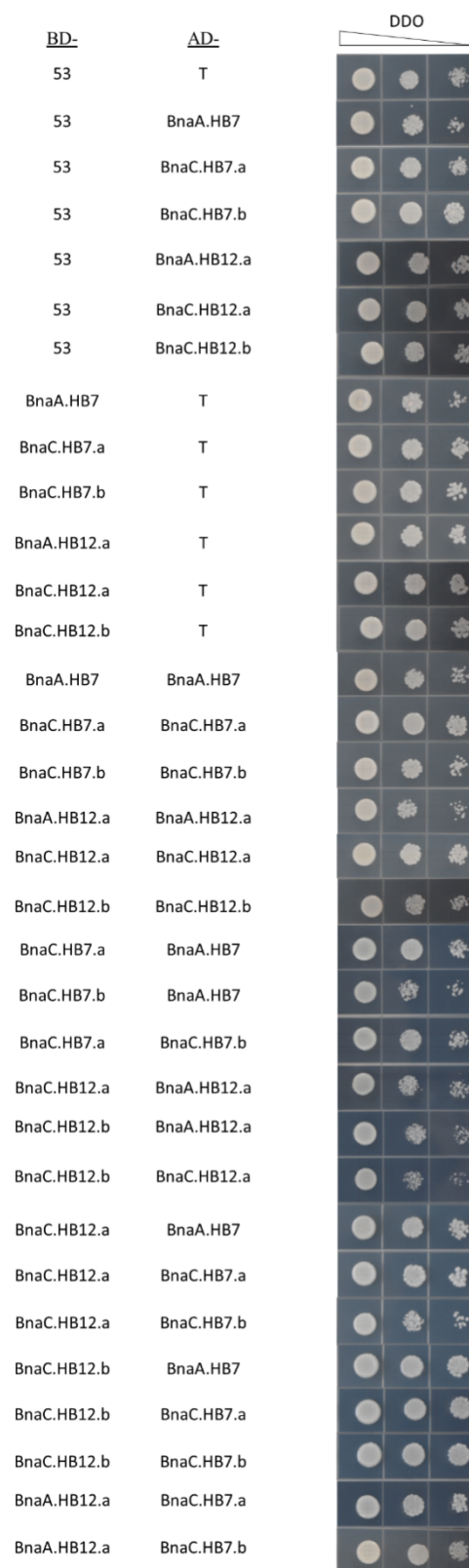

Figure S4 Growth of double carrier co-transferred yeast in DDO medium

Supplement: Supplementary file 1 [file genes-13-02139-s001.zip › Figure S4.pdf]
